# The unique N-terminal sequence of the BK_Ca_ channel α-subunit determines its modulation by β-subunits

**DOI:** 10.1371/journal.pone.0182068

**Published:** 2017-07-27

**Authors:** Ramón A. Lorca, Xiaofeng Ma, Sarah K. England

**Affiliations:** Center for Reproductive Health Sciences, Department of Obstetrics and Gynecology, Washington University in St. Louis, St. Louis, Missouri, United States of America; Indiana University School of Medicine, UNITED STATES

## Abstract

Large conductance voltage- and Ca^2+^-activated K^+^ (BK_Ca_) channels are essential regulators of membrane excitability in a wide variety of cells and tissues. An important mechanism of modulation of BK_Ca_ channel activity is its association with auxiliary subunits. In smooth muscle cells, the most predominant regulatory subunit of BK_Ca_ channels is the β1-subunit. We have previously described that BK_Ca_ channels with distinctive N-terminal ends (starting with the amino acid sequence MDAL, MSSN or MANG) are differentially modulated by the β1-subunit, but not by the β2. Here we extended our studies to understand how the distinct N-terminal regions differentially modulate channel activity by β-subunits. We recorded inside-out single-channel currents from HEK293T cells co-expressing the BK_Ca_ containing three N-terminal sequences with two β1-β2 chimeric constructs containing the extracellular loop of β1 or β2, and the transmembrane and cytoplasmic domains of β2 or β1, respectively. Both β chimeric constructs induced leftward shifts of voltage-activation curves of channels starting with MANG and MDAL, in the presence of 10 or 100 μM intracellular Ca^2+^. However, MSSN showed no shift of the voltage-activation, at the same Ca^2+^ concentrations. The presence of the extracellular loop of β1 in the chimera resembled results seen with the full β1 subunit, suggesting that the extracellular region of β1 might be responsible for the lack of modulation observed in MSSN. We further studied a poly-serine stretch present in the N-terminal region of MSSN and observed that the voltage-activation curves of BK_Ca_ channels either containing or lacking this poly-serine stretch were leftward shifted by β1-subunit in a similar way. Overall, our results provide further insights into the mechanism of modulation of the different N-terminal regions of the BK_Ca_ channel by β-subunits and highlight the extension of this region of the channel as a form of modulation of channel activity.

## Introduction

Large conductance voltage- and Ca^2+^-activated K^+^ (BK_Ca_) channels are important regulators of membrane excitability. Their activation induces repolarization of the membrane potential after depolarization in order to buffer excitatory stimulation. BK_Ca_ channels are expressed in several cell types, such as neurons [1], vascular and myometrial smooth muscle [2, 3] and secretory cells [3], where they show distinct biophysical, pharmacological and functional characteristics. This difference in activity within specific cell types may be explained by various modulatory mechanisms, such as alternative splicing [5–7], post-translational modifications [8–10], membrane microdomain localization [11–14] and association with auxiliary subunits [15–20].

BK_Ca_ channels are comprised of tetramers of α-subunits, each one containing seven transmembrane domains (S0-S6), an extracellular N-terminal region and an intracellular C-terminal domain [21]. Three possible translation initiation codons have been described in the first exon of the BK_Ca_ α-subunit [22, 24]. The extracellular extended N-terminal regions are unique among all potassium channels [21], highly conserved in mammalian BK_Ca_ channels [22, 24], and seem to be intended to isolate different initiation start sites from the main body of the channel protein by the insertion of long flexible peptides. In one case, an initiation start is isolated from the main body of the channel by a stretch of 19 glycine/serine residues; in another case, a start site is isolated by a polyserine stretch of 22 residues [22, 24]. Initially, the third start codon, which generates a protein starting with the amino acid sequence MDAL, was described as the main translation initiation site to produce functional channels [22, 24]. However, recent studies have also described BK_Ca_ channels starting at either the first and second initiation codons, proteins starting with MANG and MSSN amino acid sequence, respectively [25–27]. The significance of this unusual configuration is unknown, but it is known that one or more BK_Ca_ β-subunits interact with the N-terminal region [21, 28].

Several lines of evidence showed that the N-terminal end, the first transmembrane domain (S0) and the C-terminal region of the α-subunit are required for the interaction between α- and its auxiliary, β1-subunit [21, 28, 28]. Modulatory β-subunits have been described to provide tissue-specific modulation to the pore-forming α-subunit. The β1-subunit is widely expressed in smooth muscle cells, where it increases BK_Ca_ channel voltage-dependency and apparent Ca^2+^-sensitivity [29, 31], playing a crucial role in maintaining vascular tone [31, 33], regulating blood pressure [31, 33] and myometrial contractility [35]. The β1-subunit is an integral membrane protein containing two transmembrane domains, with a large extracellular loop and both N- and C-terminal ends cytoplasmic. The intracellular N- and C-terminal domains of the β1-subunit seem to be essential for its modulation of channel activity [36, 36], although some reports have also suggested the transmembrane domains and extracellular loop of β1 participate in this modulation [37, 38].

In a previous study, we have shown that the three different N-terminal constructs of BK_Ca_, produced by the three proposed initiation sites, are differentially modulated by the β1-subunit, an effect not seen when co-expressed with a non-inactivating β2-subunit (β2ND). Voltage-activation of the BK_Ca_ channels starting at either the first or third initiation codons, MANG and MDAL, respectively, was shifted leftward (or to hyperpolarizing potentials) when co-expressed with β1, compared to α alone, an effect not measured when β1 was co-expressed with channels starting with MSSN [39]. These results suggest that distinct N-termini might provide an additional mechanism of modulation of BK_Ca_ channel activity.

Here, we extend our previous studies in order to investigate the molecular determinants underlying the selective modulation of the different BK_Ca_ channel N-terminal constructs by the β1-subunit. Using three starting sequences (MANG, MSSN and MDAL) and two truncated forms of the α-subunit, we assessed the effects of the β1- and β2ND-subunits, and two distinct β1-β2 chimeric constructs, formed by either extracellular loop of β1 or β2 subunit, and transmembrane and intracellular domains from either β2ND or β1 (β2NDβ1β2 or β1β2β1, respectively), on single-channel BK_Ca_ currents. We observed that BK_Ca_ channels with distinct N-termini were modulated by the two chimeric constructs in a distinctive manner: the voltage-activation curve was not shifted in channels starting with MSSN when co-expressed with β2NDβ1β2. This mimicked what was seen in the presence of β1 subunit, suggesting that the extracellular loop of β1-subunit may block modulation of MSSN constructs. In addition, we observed that the presence of the poly-serine stretch located between the second and third initiation site of α-subunit was not enough to induce blocking of β1 modulation, as observed previously with MSSN [39]. These results provide evidence that BK_Ca_ channels with distinctive N-termini can be differentially modulated by β-subunits, suggesting a novel mechanism to regulate the biophysical and functional properties of the channels in different cell types.

## Materials and methods

### cDNA constructs

**Fig 1:**
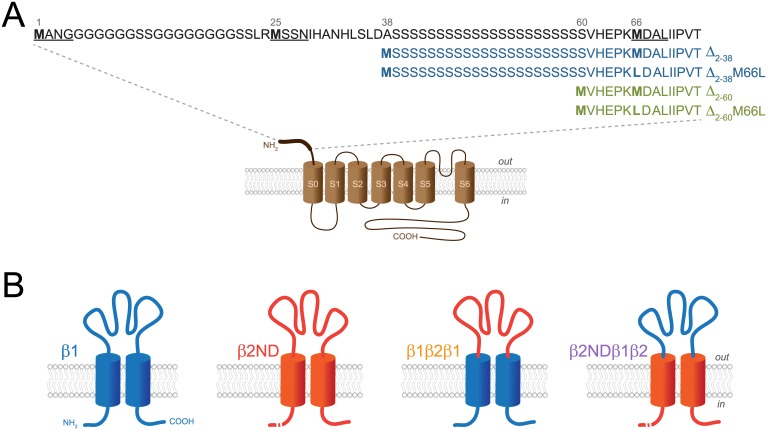
BK_Ca_ α-subunit N-terminal, β1, β2ND and β1-β2 chimeric constructs. **A**, Schematic representation of the BK_Ca_ α-subunit N-terminal sequence and truncated constructs. Names used for the N-terminal constructs are underlined in the sequence of the BK_Ca_ α-subunit. N-terminal sequences of the four truncated constructs used in this study are shown; Δ_2–38_, Δ_2-38_M66L, Δ_2–60_ and Δ_2-60_M66L. **B**, Schematic representation of BK_Ca_ β1, β2ND and β1-β2 chimeric constructs used in this study.

Human BK_Ca_ α-subunit starting at different codons (Fig 1A), MANG (GenBank Accession Number BC137137.1) and MSSN, were cloned from human uterus, MDAL was subcloned by PCR from the MANG construct, as described in a previous study [39]. Two truncated BK_Ca_ α constructs (Δ_2–38_ and Δ_2–60_) were generated by PCR using the MSSN construct as the template (Fig 1A); site-directed mutagenesis of Met 66 to Leu, in these two truncated constructs, was performed by Mutagenex, Inc. (Suwanee, GA). The cDNA encoding for the different α-subunit constructs were inserted into the pCMV site of a pBudCE4.1 plasmid vector (Invitrogen, Carlsbad, CA) containing an optimal Kozak sequence (GACCACC) upstream of the start codon and including the mCherry reporter in the EF1-α site. The cDNA encoding the human β1-subunit (GenBank Accession Number U25138.1) and a β2-subunit (GenBank Accession Number NM_181361.2) lacking the inactivating N-terminal region (Δ_2–20_, β2ND, a kind gift from Dr. Jianmin Cui, Washington University in St. Louis) were cloned into the EF1-α site of pBudCE4.1; eGFP was cloned by PCR into the pCMV site as a reporter. The β1-β2-subunit chimeric constructs were kindly provided by Dr. Christopher J. Lingle (Washington University in St. Louis) and were inserted into the EF1-α site of pBudCE4.1; eGFP was used as a reporter. Two chimeric constructs were used: one that contains both transmembrane and intracellular domains from β1-subunit and extracellular loop from β2-subunit (β1β2β1, Fig 1B), and another containing a truncated N-terminal region (Δ_2–20_, β2ND), both transmembrane domains and intracellular C-terminus of β2-subunit, and the extracellular loop from β1-subunit (β2NDβ1β2, Fig 1B). Plasmid DNAs for transfection were isolated with a Plasmid Maxi kit (Qiagen, Hilden, Germany).

### Cell culture and transfection

Human embryonic kidney (HEK) 293T cells (ATCC, Manassas, VA) were grown to 60%-80% confluency in DMEM/F12 supplemented with 10% FBS and 50 μg/ml gentamicin (all from Gibco, Carlsbad, CA). Cells were transiently transfected with constructs expressing the human BK_Ca_ channel with different N-terminal ends. Another set of cells were co-transfected with the BK_Ca_ α-subunit constructs and either the β1-subunit, β2ND, β1β2β1 or β2NDβ1β2 constructs (in a 1:4 α:β molar ratio). All transfections were performed using Lipofectamine 2000 reagent (Invitrogen) according to the manufacturer’s directions. Cells were used in subsequent experiments 24–48 h post-transfection.

### Electrophysiology

Single-channel recordings in the inside-out configuration were performed at room temperature in a bath solution containing (in mM): 140 KCl, 20 KOH, 10 HEPES, and either 5 (H)EDTA and 0.1–10 μM free-Ca^2+^, or 5 EGTA and 100 μM free-Ca^2+^ (pH 7.2 with HCl). Free-Ca^2+^ concentration (0.1–10 μM) was measured using a Ca^2+^-sensitive electrode (Thermo Fisher Scientific, Waltham, MA). Pipette solution contained (in mM): 140 KCl, 20 KOH, 2 MgCl_2_, and 10 HEPES (pH 7.4). Single-channel currents were recorded at a sampling rate of 100 kHz and filtered at 5 kHz by using an Axopatch 200B amplifier (Molecular Devices, Sunnyvale, CA). Currents were evoked with 10 mV voltage-steps (1000-ms duration) from -160 to +200 mV, from a holding potential of 0 mV by using pCLAMP 10 software (Molecular Devices). This protocol was repeated at least three times on each patch. For analysis purposes, recordings on the same patch were concatenated, attaining at least a 3-s length recording from each voltage-pulse. Mean open probability (*P*_*o*_) values were calculated by using pCLAMP 10 software. Patches containing three or fewer channels were used for *P*_*o*_ analysis. *P*_*o*_ was plotted against membrane potential (*V*) and fit to a Boltzmann function, *P*_*o*_ = *P*_*o* (max)_ / (1 + *e*−^*zF (V–V0*.*5) / RT*^), in order to determine half-maximal activation voltage (*V*_*0*.*5*_) values for each experiment. This analysis was performed with Graph Pad software (San Diego, CA). For analysis of open and closed dwell-times of the channel (MANG, MSSN, or MDAL), in the presence or absence of β2ND, β1β2β1 or β2NDβ1β2, inside-out patches containing only one channel were held at membrane potentials ranging between -100 mV and -20 mV, depending on the level of activation, for 3 min with 10 μM Ca^2+^ in the bath. Open and closed dwell-times histograms were plotted in log-bin timescales and fit with double (or single as indicated) Gaussian functions to obtain time constants (τ) and relative distribution (P) of the data under the curve by using pCLAMP 10 software. To ensure a clear estimation of τ, only recordings with a *P*_*o*_ value less than 0.8 were included in the analysis. All the recordings were performed and analyzed with the construct composition blinded to the investigator.

### Statistical analysis

Data obtained were subjected to either non-parametric Mann-Whitney U-test or two-way ANOVA followed by Sidak’s multiple comparison test (Graph Pad software). A *P* value < 0.05 was considered significant. All data are presented as mean ± S.E.M.

## Results

### BK_Ca_ β1-β2 chimeric constructs modulate α-subunit with distinct N-termini

We have previously shown that BK_Ca_ α-subunit with distinct N-termini are differentially modulated by the β1-subunit; BK_Ca_ channels starting with MSSN showed a complete lack of β1 modulation, whereas the voltage activation curves of channels starting with MANG and MDAL were shifted to the left in the presence of β1-subunit [39]. Interestingly, the voltage activation curves in all three N-terminal constructs were shifted by the β2-subunit lacking its inactivation sequence (β2ND) [39]. To evaluate the region of the β1-subunit responsible for blocking its modulation of MSSN, we used two chimeric constructs (Fig 1B). One is composed of the transmembrane and intracellular domains of β1 and the extracellular loop of β2 (β1β2β1) and the other is composed of the transmembrane and intracellular domains of β2 and the extracellular loop of β1 (β2NDβ1β2). We expressed all constructs with the BK_Ca_ channel and recorded single-channel currents in the inside-out configuration, at different intracellular Ca^2+^ concentrations ([Ca^2+^]_i_). These different [Ca^2+^]_i_ were used to dissect the allosteric increase of the open probability of the channel mediated by Ca^2+^ from the effect of voltage.

**Fig 2:**
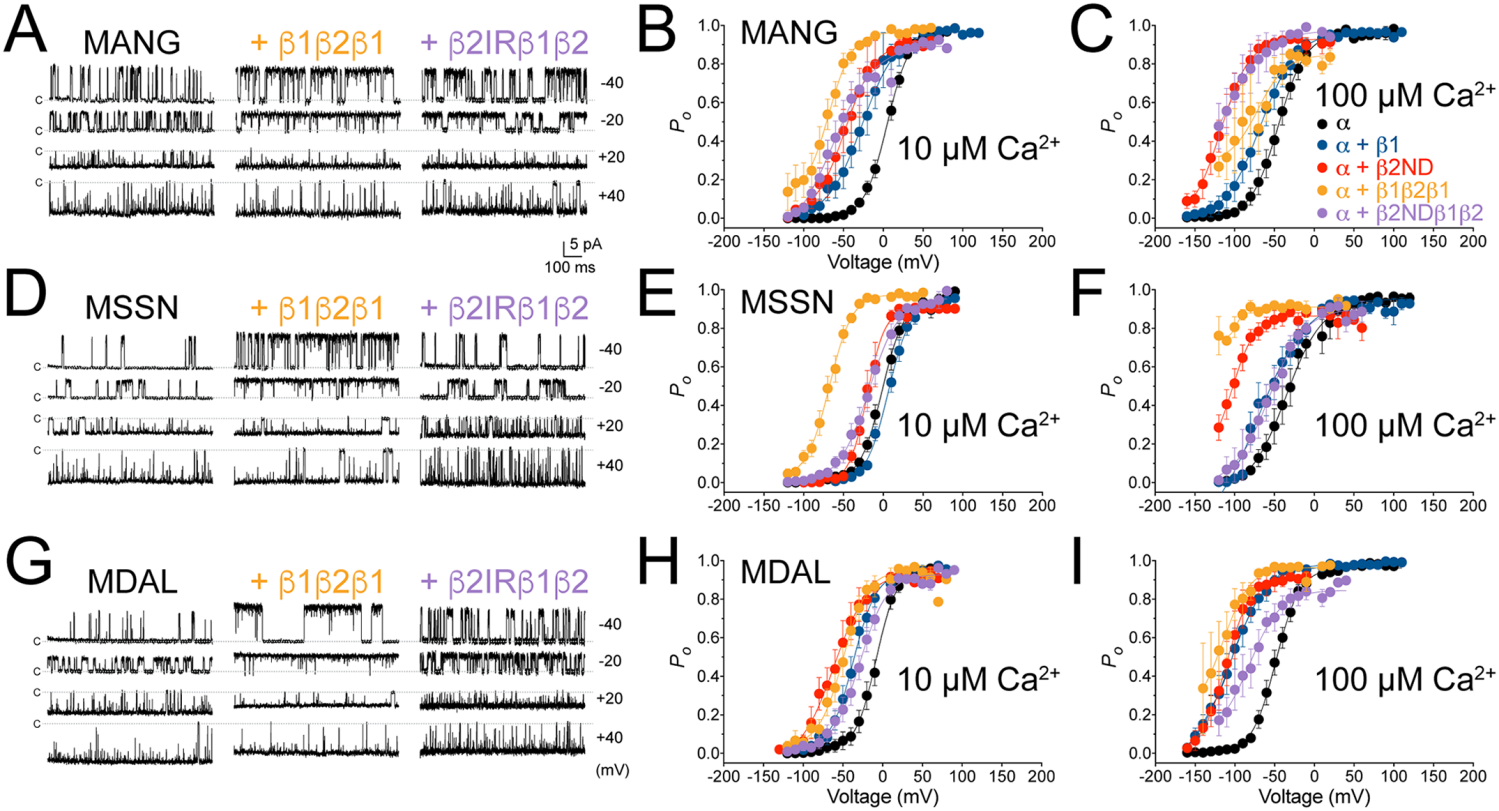
BK_Ca_ α-subunit with distinct N-termini are differentially modulated by β1-β2 chimeric constructs. Representative inside-out single-channel recordings from HEK293T cells transfected with MANG (**A**), MSSN (**D**) or MDAL (**G**) in the presence or absence of β1β2β1 or β2NDβ1β2, at different membrane potentials (-40 mV to +40 mV) with 10 μM Ca^2+^ in the bath. Dashed lines indicate closed (C) states of the channels. Voltage-activation of MANG (**B** and **C**), MSSN (**E** and **F**) or MDAL (**H** and **I**), in the absence (α, black symbols) or presence of β1 (blue symbols), β2ND (red symbols), β1β2β1 (orange symbols) or β2NDβ1β2 (purple symbols), expressed as open probability (*P*_*o*_) of the channel, in the presence of 10 μM (**B**, **E** and **H**) or 100 μM (**C**, **F**, and **I**) Ca^2+^ in the bath; n = 3–19, symbols are mean ± SEM.

**Fig 3:**
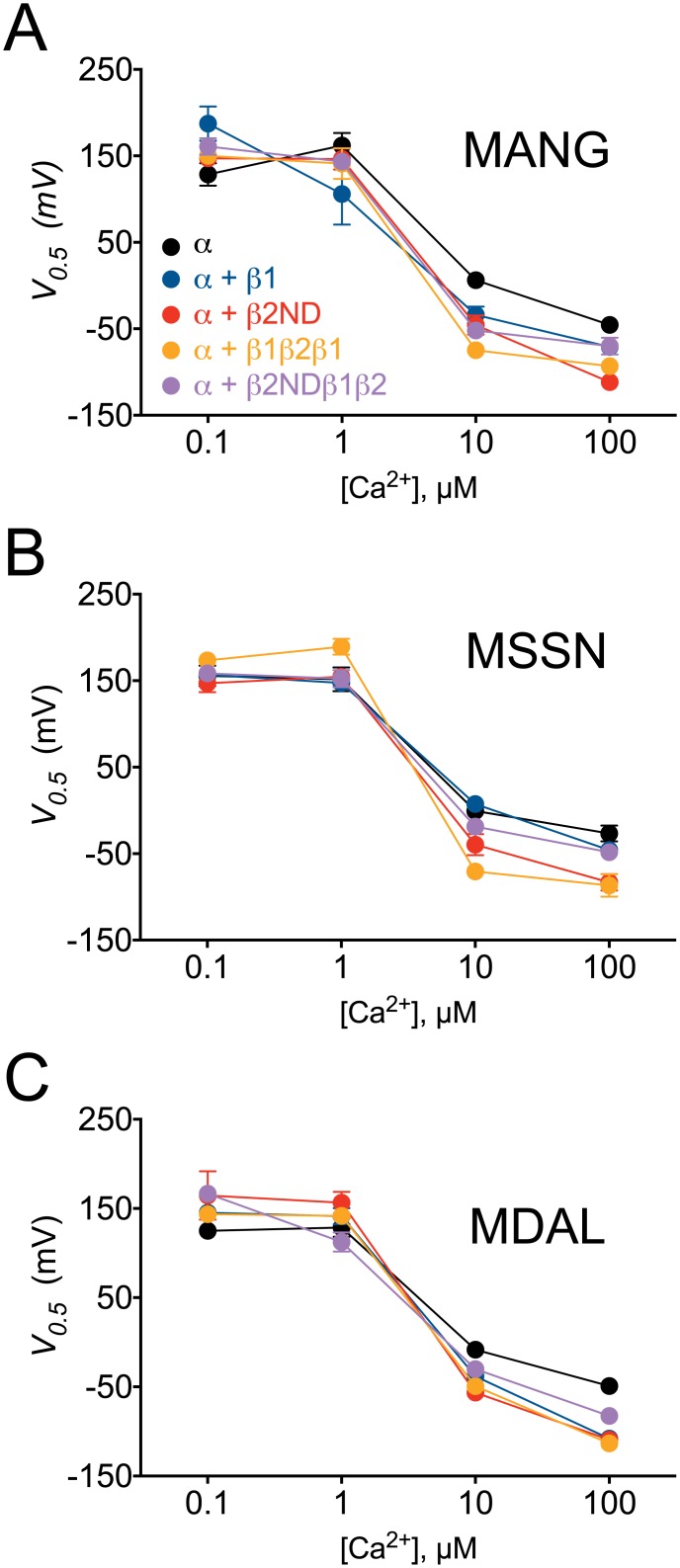
Modulation of voltage-dependent activation in BK_Ca_ α-subunit with distinct N-termini by β-subunit constructs. Analysis of the voltage of half maximal activation (*V*_*0*.*5*_) of the N-terminal BK_Ca_ constructs MANG (**A**), MSSN (**B**) or MDAL (**C**), in the absence (α, black symbols) or presence of β1 (blue symbols), β2ND (red symbols), β1β2β1 (orange symbols) or β2NDβ1β2 (purple symbols), at different [Ca^2+^] in the bath. Symbols are mean ± SEM.

At low [Ca^2+^]_i_, 0.1 μM, the voltage activation curve of MANG was shifted to the right by the β1-subunit, but not significantly by β2ND or the chimeric constructs, whereas the MDAL voltage activation curve was shifted to the right by β2ND and β2NDβ1β2, but not by β1 or β1β2β1 (S1 Table). Channels starting with MSSN construct were not affected by β-subunits or the chimeric constructs at 0.1 or 1 μM Ca^2+^ (S1 Table). Neither MANG nor MDAL curves were significantly changed by any β-subunit at 1 μM Ca^2+^ (S1 Table). Interestingly, at 10 μM [Ca^2+^]_i_, both chimeric constructs (β1β2β1 and β2NDβ1β2) induced significant leftward shifts in the voltage-activation curves in cells expressing MANG. This shift was similar to those observed with β2ND, but larger than with the β1, whereas only β2ND and β1β2β1 induced significant leftward shifts in the MANG construct at 100 μM Ca^2+^ (Figs 2A–2C and 3A, S1 Table). The voltage activation of the MSSN construct, however, was leftward shifted in the presence of either β2ND or β1β2β1, but not by β1 or β2NDβ1β2, at both 10 and 100 μM Ca^2+^ (Figs 2D–2F and 3B, S1 Table). Finally, the MDAL construct activation curve was shifted to the left in the presence of all β-subunits tested at 100 μM Ca^2+^, and by β1, β2ND and β1β2β1 at 10 μM Ca^2+^ (Figs 2G–2I and 3C, S1 Table).

### Modulation of BK_Ca_ kinetics by β2ND and β1-β2 chimeric constructs

**Fig 4:**
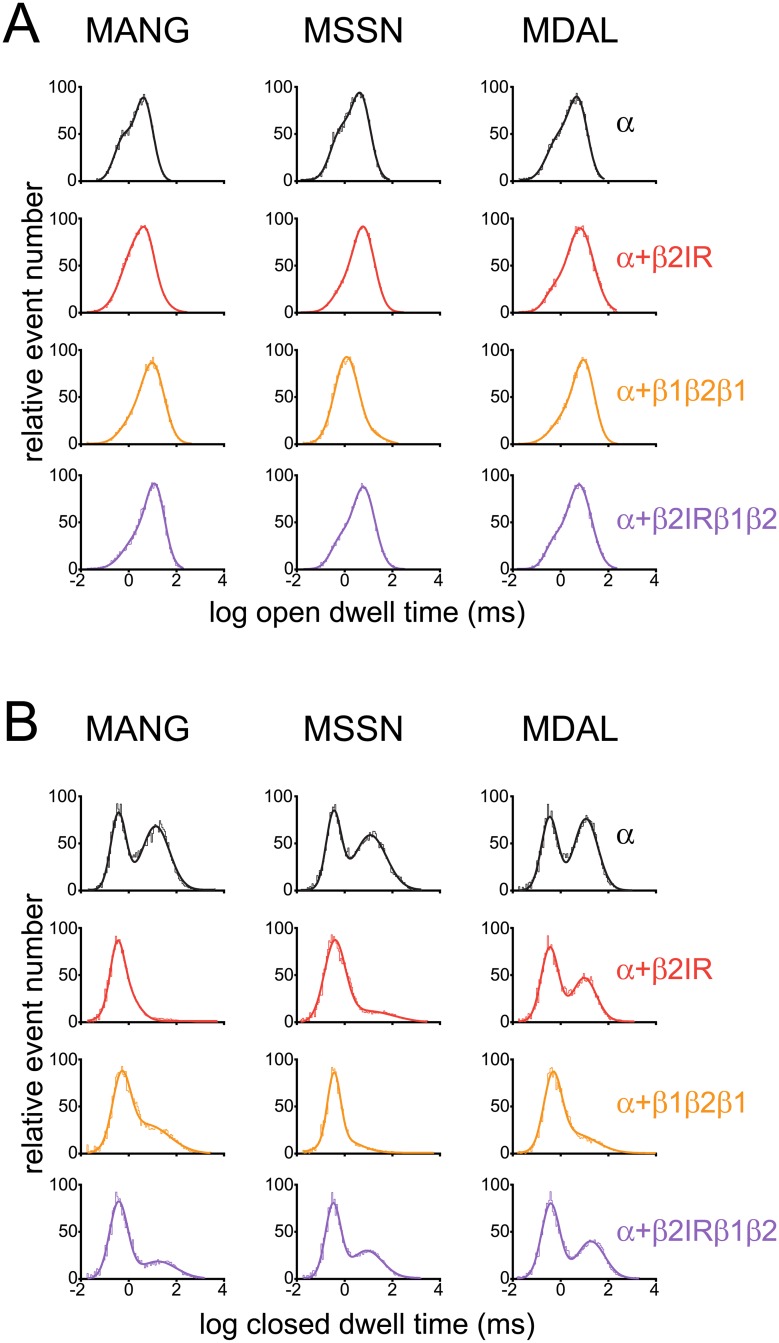
Effects of β2ND and β1-β2 chimeric constructs on BK_Ca_ α-subunit N-terminal constructs single-channel kinetics. Open (**A**) and (**B**) closed dwell-times distribution histograms of single-channels in HEK293T cells expressing MANG, MSSN or MDAL constructs. Patches containing channels composed by α-subunit alone (black lines), α+β2ND (red lines) α+β1β2β1 (orange lines) or α+β2β1β2 (purple lines) were analyzed (n = 6–11). Single-channel currents were elicited by holding the membrane potential at a certain voltage (-20 mV to -100 mV) for at least 1 min in the presence of 10 μM Ca^2+^ in the bath. Histograms were plotted in log-bin timescales and fitted with double exponential functions.

Because β1 and β2 subunits modulate the BK_Ca_ channel by different mechanisms [40], we investigated the effect of β2ND and the β1/β2 chimeric constructs on the kinetics of the BK_Ca_ α-subunit N-terminal constructs. Initially, we investigated the open-state kinetics. We found that β2ND significantly increased the τ1 and τ2 of open state of MANG (3.2- and 0.4-fold, respectively, Fig 4A and Table 1), whereas it increased only τ2 in MSSN (0.3-fold, Fig 4A and Table 1). Conversely, although only τ1 was decreased by β2ND in MDAL, the proportion of these populations was shifted from τ1 towards τ2 (Fig 4A and Table 1). In MANG, both β1β2β1 and β2NDβ1β2 significantly increased τ2 (1.6- and 2-fold, respectively, Fig 4A and Table 1), whereas only β2NDβ1β2 increased τ2 in MDAL (1.2-fold, Fig 4A and Table 1). Surprisingly, in MSSN, τ2 was redistributed by β1β2β1 towards shorter values (τ1, Table 1), revealing an overall reduction in open dwell-time histograms (Fig 4A). In turn, β2NDβ1β2 showed an increase in τ2 of MSSN comparable to that observed with β2ND (Fig 4A and Table 1).

**Table 1:** Effect of β2ND,β1β2β1 and β2NDβ1β2 constructs on the open dwell-times of BK_Ca_ channels with distinctive N-terminal regions.

	Open, mean ± SEM			
Construct	α	+β2ND	+β1β2β1	+β2NDβ1β2
MANG				
τ_1_	0.59 ± 0.05	2.50 ± 0.06*	1.59 ± 0.81	2.58 ± 1.63
P_1_	0.35 ± 0.04	0.88 ± 0.02	0.26 ± 0.11	0.58 ± 0.26
τ_2_	4.31 ± 0.19	6.11 ± 0.20*	11.06 ± 1.19*	13.06 ± 1.05*
P_2_	0.65 ± 0.03	0.12 ± 0.02	0.74 ± 0.11	0.42 ± 0.23
MSSN				
τ_1_	0.64 ± 0.08	0.79 ± 0.14	3.09 ± 0.26*	0.65 ± 0.05
P_1_	0.37 ± 0.05	0.19 ± 0.04	0.52 ± 0.06	0.22 ± 0.02
τ_2_	4.82 ± 0.31	6.32 ± 0.27*	3.30 ± 0.30*	6.35 ± 0.19*
P_2_	0.63 ± 0.05	0.81 ± 0.04	0.48 ± 0.06	0.78 ± 0.02
MDAL				
τ_1_	0.80 ± 0.15	0.45 ± 0.05*	2.34 ± 1.76	0.50 ± 0.04*
P_1_	0.41 ± 0.07	0.11 ± 0.02	0.39 ± 0.20	0.16 ± 0.02
τ_2_	5.63 ± 0.51	6.38 ± 0.18	12.24 ± 0.64*	6.05 ± 0.17
P_2_	0.59 ± 0.06	0.89 ± 0.02	0.61 ± 0.18	0.84 ± 0.02

Time constants (τ_1_ and τ_2_) are expressed in milliseconds. P_1_ and P_2_ are relative distributions of data under curves used to fit the results shown in Fig 4.

* *P* < 0.05 compared to α-subunit, non-parametric Mann-Whitney U-test.

The most remarkable effect of β2ND was on the relative distribution of closed dwell-times, τ1 and τ2, of MANG and MSSN, increasing the proportion of shorter, τ1, closed dwell-times (P_1_) and reducing the proportion of longer, τ2, closed dwell-times (Fig 4B and Table 2), in the case of MANG, both closed τ1 and τ2 overlapped in the presence of β2ND and their values were close to those observed for τ1 in MANG alone (Fig 4B and Table 2), indicating that both MANG and MSSN spent less time in the closed state in the presence of β2ND. Similarly, β1β2β1 redistributed the closed dwell-times of MSSN towards shorter values, however, that redistribution was less evident with β2NDβ1β2 (Fig 4B and Table 2). Finally, the β2NDβ1β2 construct reduced the relative distribution of closed dwell-times towards shorter values in MANG and, to a lesser extent, in MDAL constructs (Fig 4B). Overall, these results both confirm the idea that the β2-subunit stabilize the BK_Ca_ channel in the open state, and suggest that the extracellular loop of β1-subunit might be responsible to maintain MSSN channels in the closed state.

**Table 2:** Effect of β2ND,β1β2β1 and β2NDβ1β2 constructs on the closed dwell-times of BK_Ca_ channels with distinctive N-terminal regions.

	Closed, mean ± SEM			
Construct	α	+β2ND	+β1β2β1	+β2NDβ1β2
MANG				
τ_1_	0.38 ± 0.01	0.33 ± 0.01*	0.50 ± 0.01*	0.40 ± 0.01*
P_1_	0.40 ± 0.01	0.49 ± 0.08	0.50 ± 0.06	0.65 ± 0.02
τ_2_	13.86 ± 0.43	0.66 ± 0.08*	6.04 ± 2.10*	15.02 ± 1.31
P_2_	0.60 ± 0.01	0.51 ± 0.08	0.50 ± 0.12	0.34 ± 0.03
MSSN				
τ_1_	0.36 ± 0.01	0.40 ± 0.01*	0.36 ± 0.005	0.32 ± 0.01*
P_1_	0.39 ± 0.01	0.72 ± 0.08	0.66 ± 0.03	0.57 ± 0.02
τ_2_	12.48 ± 0.39	9.10 ± 0.98*	0.79 ± 0.13*	8.33 ± 0.62*
P_2_	0.61 ± 0.02	0.28 ± 0.21	0.34 ± 0.04	0.43 ± 0.03
MDAL				
τ_1_	0.35 ± 0.01	0.36 ± 0.01	0.46 ± 0.01*	0.38 ± 0.01*
P_1_	0.41 ± 0.01	0.55 ± 0.01	0.61 ± 0.04	0.57 ± 0.01
τ_2_	11.35 ± 0.29	9.8 ± 0.39*	5.74 ± 1.62*	18.32 ± 0.77*
P_2_	0.59 ± 0.02	0.45 ± 0.02	0.39 ± 0.06	0.43 ± 0.02

Time constants (τ_1_ and τ_2_) are expressed in milliseconds. P_1_ and P_2_ are relative distributions of data under curves used to fit the results shown in Fig 4.

* *P* < 0.05 compared to α-subunit, non-parametric Mann-Whitney U-test.

### Lack of modulation by β1 in MSSN is independent of the presence of poly-serine stretch

**Fig 5:**
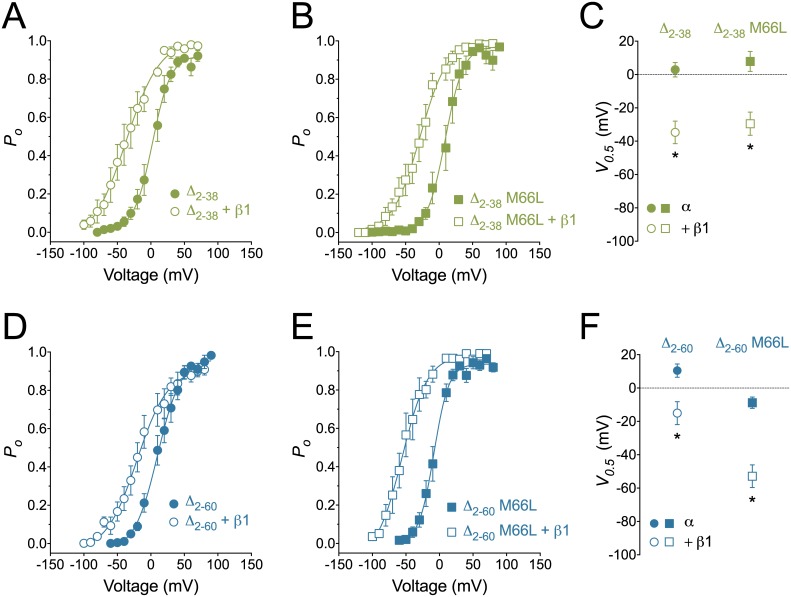
BK_Ca_ α-subunit N-terminal truncated constructs are similarly modulated by the β1-subunit. Voltage dependence of BK_Ca_ activation of two truncated forms of the BK_Ca_ α-subunit; lacking amino acids 2–38 (**A** and **B**, Δ_2–38_ and Δ_2-38_M66L) or amino acids 2–60 (**D and E**, Δ_2–60_ and Δ_2-60_M66L), in the presence (open symbols) or absence (closed symbols) of the β1-subunit, expressed as open probability of the channel (*P*_*o*_), in the presence of 10 μM Ca^2+^ in the bath; n = 5–10, symbols are mean ± SEM. **C** and **F**, Analysis of the voltage of half maximal activation (*V*_*0*.*5*_) between the N-terminal truncated constructs alone (closed symbols) and in the presence of β1 (open symbols). Symbols are mean ± SEM, * *P* < 0.05 compared to α.

Because, in our previous study, the BK_Ca_ channel starting at MSSN was not altered by the β1-subunit [39], we focused on the structural determinants of this N-terminal region responsible for the reduced modulation. We evaluated the role of the poly-serine stretch located between residues 39 and 60 within the N-terminal region of BK_Ca_ α-subunit (Fig 1A) by using two truncated constructs of MSSN: one containing only the poly-serine stretch (Δ_2–38_, Fig 1A) or one lacking the poly-serine stretch (Δ_2–60_, Fig 1A). We observed that β1-subunit induced significant leftward shifts in the voltage-activation curves in Δ_2–38_ (from 2.9 ± 4.3 mV to -34.7 ± 6.8 mV, *P* < 0.01, Fig 5A and 5C) and Δ_2–60_ (from 10.5 ± 4 mV to -15 ± 6.9 mV, *P* < 0.01, Fig 5D and 5F), suggesting that the poly-serine stretch does not participate in the lack of modulation by β1 in MSSN. To confirm that the effects observed in these two α-subunit truncated forms were not due to expression of the downstream MDAL (third initiation site) we mutated Met-66 to Leu. The voltage activation curves of both constructs, Δ_2-38_M66L and Δ_2-60_M66L, were significantly shifted to the left by the β1-subunit (from 7.8 ± 5.9 mV to -29.5 mV and from -8.8 ± 3.4 mV to -52.9 ± 6.8 mV, respectively, *P* < 0.01, Fig 5B, 5C, 5E and 5F). Altogether, these results suggest that the poly-serine stretch located within the N-terminal region of α-subunit is not responsible for the reduced modulation by β1 observed in MSSN.

## Discussion

The BK_Ca_ channel plays a major role in buffering excitation in a wide variety of cells and tissues. The interaction of the pore-forming α-subunit with different modulatory subunits is an important mechanism to regulate the activity of this channel [17, 18, 20], providing tissue-specificity of BK_Ca_ activation. Here we focused on the differential effect of the modulatory β1-subunit in the activation of distinct N-termini of the α-subunit as previously described [39]. We extended these studies by using truncated constructs of the α-subunit or chimeric constructs of the β-subunits to determine the structural determinants within the distinctive N-terminal region of the α-subunit or extracellular loop of β-subunits responsible for this differential modulation. In the present study, we found two main observations; first, that the extracellular loop of the β1-subunit underlies the lack of modulation of MSSN, and second, that the poly-serine stretch region within the N-terminal domain of the α-subunit is not enough to dampen the modulation of MSSN by the β1-subunit.

The unique extracellular N-terminal region of the BK_Ca_ channel is highly conserved among mammals [22, 24], but it differs in other organisms such as chicken [41], *Drosophila melanogaster* [21, 42] or *Caenorhabditis elegans* [44], and it is exclusively found only in BK_Ca_ channels of the voltage-gated K^+^ channel family [21]. The extracellular N-terminal region, the S0 domain, and the intracellular C-terminal domain of the BK_Ca_ α-subunit have been described to participate in the interaction with the β1-subunit [21, 28, 28]. We have shown that this distinctive N-terminal region, as is found in BK_Ca_ channels starting with MSSN, reduced the modulation of voltage activation by β1 [39]. The N-terminal region contains an intriguing sequence including a poly-serine stretch between Ala-38 and Val-61 (Fig 1A), which has been proposed to be responsible for blocking Ca^2+^-sensitivity of MSSN modulation by the β1-subunit [25]. We evaluated whether the poly-serine stretch was enough to block the β1-subunit modulation of the BK_Ca_ channel; two truncated constructs of the BK_Ca_ α-subunit were tested, one lacking the poly-serine stretch and starting from residue Val-61 and another containing the poly-serine stretch, starting from Ser-39, and lacking the residues between Met-25 and Ala-38 (Fig 1A). The voltage activation curves of both truncated constructs were shifted to the left by β1, suggesting that the poly-serine stretch is not responsible in reducing the modulation of MSSN by β1. Poly-serine stretches are predicted to be low complexity, highly disordered structures [45, 45]. The function of poly-serine regions in eukaryotic proteins is not well understood, however, in prokaryotic proteins, they are predicted to act as flexible linkers between functional domains [45], thus, it is possible that the poly-serine stretch is functioning as a linker between the channel and its N-terminal domain. Interestingly, the sequence comprised between Met-25 and Ala-38, right before the poly-serine stretch, is also highly conserved among mammals. Further studies, either by adding a soluble peptide containing this region, as shown for the BK_Ca_ β3 subunit [46], or by mutation of key residues within this region might directly elucidate the role of this region or the residues responsible in dampening β1 modulation. Although all the truncated constructs tested were modulated by β1, the Δ_2-60_M66L construct seems to be shifted to the left even in the absence of β1 when compared to MDAL or Δ_2–60_. A further leftward shift was observed in the presence of β1, which could be due to the expression of MDAL in Δ_2–60_, which we cannot exclude as a possibility in this construct, or to a structural change induced by the M66L point mutation in proximity to the initiation codon.

Our previous results showing that MSSN construct is not modulated by β1 [39] together with the present observations showing that the chimeric construct containing β1 extracellular loop (β2NDβ1β2) did not modulate MSSN demonstrate that this region of the β1-subunit plays an important role in the lack of modulation of this construct. However, we cannot exclude that the transmembrane domains and/or cytoplasmic regions could also participate. Interestingly, in the MSSN construct, β1β2β1 seems to enhance the leftward shift induced by β2ND alone, suggesting that the β1 transmembrane domains and/or cytoplasmic regions could be acting in a synergistic way with the β2 extracellular loop to induce further activation of MSSN. Further studies using β1-β2 chimeric constructs switching either the transmembrane and/or cytoplasmic domains of these subunits would help to elucidate the role of these regions in the modulation of MSSN, as shown by others for the MDAL construct [36, 36, 38]. In addition, MSSN was not modulated by any β-subunit in the presence of low [Ca^2+^]_i_ (0.1 and 1 μM), whereas higher [Ca^2+^]_i_ (10 and 100 μM) showed a differential modulation by β1 and β2ND, suggesting that this differential modulation depends on the presence of Ca^2+^.

The β1- and β2-subunits have been proposed to modulate the BK_Ca_ channel kinetics through independent mechanisms [40], accordingly, in our studies using the MDAL construct, we have found distinct effects of β1 and β2ND on the kinetics of the BK_Ca_ channels; β1 induces a shortening of the closed dwell-times [39], whereas the effect on the closed dwell-times is less marked in the presence of β2ND. Several reports have shown that the N- and C-termini and the transmembrane domains of β1-subunit are responsible for its interaction with the BK_Ca_ α subunit [36, 36, 38]. Our results showing a comparable change in the distribution of the closed dwell-times of MDAL by either β1 or β1β2β1 constructs agree with those studies [39]. In addition, the effect of β2NDβ1β2 on the closed dwell-times of MDAL was similar to that observed with β2ND (Fig 3C). Furthermore, both the MSSN and MANG constructs showed redistribution towards shorter closed dwell-times with both the β2ND and β1β2β1. This change in proportion of closed dwell-times was less evident in the presence of β2NDβ1β2, and similar to what was observed with β1 [39]. Altogether, these analyses revealed a role for the extracellular loop of β1 in the alteration of the kinetics of MSSN.

In summary, we found that the extracellular region of β1-subunit is important in inhibiting β1-induced modulation of BK_Ca_ α-subunit starting with MSSN. Additionally, within the N-terminal sequence of BK_Ca_ α-subunit, the region between the second initiation site (Met-25) and the poly-serine stretch might contribute to the reduced modulation by β1. These mechanisms could provide additional regulation of BK_Ca_ channel activity, besides the expression of different auxiliary subunits.

## Supporting information

S1 Table Effect of β1, β2ND-subunits and β1-β2 chimeric constructs on the voltage- and Ca^2+^-activation of different BK_Ca_ channel α-subunit N-terminal constructs. Data are mean values ± SEM, number of patches are in brackets. *Italicized* values were either reproduced or complemented from Lorca *et al*. (2014) [39] and presented for comparison. * *P* < 0.05 compared to α alone.
(DOCX)
