# Supplementary material for: The unique N-terminal sequence of the BKCa channel α-subunit determines its modulation by β-subunits
Source: PLoS One. 2017 Jul 27;12(7):e0182068. doi: 10.1371/journal.pone.0182068 (PMC5531486; doi:10.1371/journal.pone.0182068)
Supplement: S1 Table — Data are mean values ± SEM, number of patches are in brackets. Italicized values were either reproduced or complemented from Lorca et al. (2014) [39] and presented for comparison. * P < 0.05 compared to α alone. (DOCX) [file pone.0182068.s001.docx]

**S1 Table. Effect of β1, β2ND-subunits and β1-β2 chimeric constructs on the voltage- and Ca^2+^-activation of different BK_Ca_ channel α-subunit N-terminal constructs.**

|  |  | **Half-maximal activation voltage (*V_0.5_*), mV** | | | | |
| --- | --- | --- | --- | --- | --- | --- |
| **Construct** | **[Ca^2+^]_i_, µM** | **α** | **+ β1** | **+ β2ND** | **+ β1β2β1** | **+ β2NDβ1β2** |
| MANG | 0.1 | 128.6 ± 13  [6] | 187.3 ± 19.9* [10] | 147.2 ± 8.8  [6] | 150 ± 4.1  [5] | 160.9 ± 9.6  [7] |
|  | 1 | 162 ± 14.5  [6] | 106.3 ± 35.4*  [5] | 146.5 ± 12.2  [7] | 141 ± 17.9  [7] | 143.4 ± 8.6  [6] |
|  | 10 | *6.3 ± 2.4*  *[18]* | *-33.9 ± 9.6**  *[10]* | *-45.7 ± 11**  *[6]* | -74.8 ± 5.7* [11] | -51.9 ± 8*  [14] |
|  | 100 | *-45.6 ± 5.9*  *[10]* | *-71 ± 8.7*  *[11]* | *-111.8 ± 6.4* [6]* | -93.3 ± 8.6*  [7] | -70 ± 9.7  [5] |
| MSSN | 0.1 | 155.5 ± 11.9 [5] | 156.7 ± 5.1  [6] | 147 ± 10.3  [8] | 173.5 ± 8.5  [4] | 158.5 ± 8.9  [5] |
|  | 1 | 151.6 ± 13.7 [6] | 147.4 ± 7.6  [6] | 154.6 ± 7.8  [7] | 189.3 ± 9.3*  [4] | 152.4 ± 10  [8] |
|  | 10 | -0.6 ± 3.1  [17] | *7.3 ± 3*  *[8]* | *-39.5 ± 12.3* [6]* | -70.5 ± 3.9* [14] | -18.6 ± 5  [15] |
|  | 100 | *-26.7 ± 9.2 [10]* | *-45.8 ± 6.4*  *[12]* | *-83.1 ± 9.6* [12]* | -86.8 ± 13.1* [3] | -48.3 ± 7.1  [7] |
| MDAL | 0.1 | 125 ± 6.9  [8] | 145.2 ± 3.1  [4] | 164.6 ± 27.1* [4] | 143.6 ± 4.7  [8] | 166.7 ± 7.6* [6] |
|  | 1 | 128.6 ± 10.3 [10] | 141.3 ± 9.1  [7] | 156.5 ± 12.2* [8] | 141.7 ± 5.7 [11] | 112.4 ± 10.7 [5] |
|  | 10 | -8.3 ± 5.2  [10] | *-38.3 ± 4.8* [10]* | *-56.4 ± 8.1* [10]* | -49.2 ± 5.3* [16] | -30 ± 5.4  [15] |
|  | 100 | *-49 ± 3.6*  *[9]* | *-108.1 ± 8**  *[9]* | *-109.3 ± 7.1* [5]* | -113.3 ± 5.5* [5] | -82.5 ± 7.4*  [7] |

Data are mean values ± SEM, number of patches are in brackets. *Italicized* values were either reproduced or complemented from Lorca *et al.* (2014) [40] and presented for comparison. * *P* < 0.05 compared to α alone.
